# Insights Into mRNA and Long Non-coding RNA Profiling RNA Sequencing in Uterus of Chickens With Pink and Blue Eggshell Colors

**DOI:** 10.3389/fvets.2021.736387

**Published:** 2021-10-07

**Authors:** Siyu Chen, Kecheng Chen, Jiaming Xu, Fangwei Li, Jinlong Ding, Zheng Ma, Gen Li, Hua Li

**Affiliations:** ^1^Guangdong Provincial Key Laboratory of Animal Molecular Design and Precise Breeding, Key Laboratory of Animal Molecular Design and Precise Breeding of Guangdong Higher Education Institutes, School of Life Science and Engineering, Foshan University, Foshan, China; ^2^Guizhou Changshun Tiannong Green Shell Laying Hen Industrial Co. Ltd, Chang Shun City, China

**Keywords:** color deposition, lncRNAs, mRNA, uterus, chicken

## Abstract

The blue egg is both of biological interest and economic importance for consumers, egg retailers, and scientists. To date, the genetic mechanisms underlying pigment have mainly focused on protein-coding genes. However, the underpinning mechanism of non-coding RNAs on the pigment deposition among different eggshell colors remains unknown. In this study, RNA sequencing was employed to profile the uterine gland transcriptome (lncRNA and mRNA) of 15 Changshun blue eggshell layers, to better understand the genetic mechanisms of deposition of blue eggshell color. Results showed that differentially expressed mRNAs, GO terms, and KEGG pathways among pink-eggshell and blue-eggshell chickens were mainly targeting immune- and transporter-related terms with the SLC family, *IgJ*, CD family, and *MTMR* genes. Furthermore, the progesterone-mediated oocyte maturation and cortisol synthesis and secretion pathway with targeted gene *PGR* and *Pbx1* were significantly enriched between blue- and pink-eggshell chickens. Integrating analysis of lncRNA and mRNA profiles predicted 4 and 25 lncRNA–gene pairs by antisense and cis analysis. They were relative to immune, nerve, and lipids and amino acid metabolisms, porphyrin, and chlorophyll metabolism with targeted gene *FECH* and oxidative phosphorylation and cardiac muscle contraction pathways with targeted gene *COX6A1*. Within blue-eggshell chickens, the GO terms hindbrain tangential cell migration and phosphatidylinositol monophosphate phosphatase activity with targeted gene *Plxna2* and *MTRM1* were identified. Integrating analysis of lncRNA and mRNA profiles predicted 8 and 22 lncRNA–gene pairs. Most pathways were mainly enriched on lipid-related metabolisms as found in mRNA sequencing. The lncRNAs did exert similar functions in color formation by modulating pigment disposition and immune- and lipid-related metabolisms. Our results provide a catalog of chicken uterine lncRNAs and genes worthy of further studies to understand their roles in the selection for blue eggshell color layers.

## Introduction

The blue egg is both of biological interest and economic importance for consumers, egg retailers, and scientists. A series of studies have been conducted on the nature of the shell pigments, the biochemical and physiological processes in various avian species involved in pigment formation, and its deposition in and on the shell (1, 2). It has been long known that the primary avian eggshell pigments are protoporphyrin IX, biliverdin IX, and biliverdin IX zinc chelate in both wild birds and poultry (3, 4). The pink, light red, and brown eggshell colors are involved with the deposition of protoporphyrin IX, while blue and green-blue eggshell colors are associated with that of biliverdin IX and biliverdin IX zinc chelate.

To date, previous studies on the genetic mechanisms underlying pigment deposition have mainly focused on protein-coding genes. The gene solute carrier organic anion transporter family member 1B3 (*SLCO1B3*) is known to be responsible for a causative mutation for the blue eggshell phenotype and is specifically expressed in the uterus, not in the other organs in chickens (5). However, the underpinning mechanism of non-coding RNAs on the pigment deposition remains unknown. A major reason is that the functional annotation of long non-coding RNAs (lncRNAs) is largely missing. LncRNAs comprise a heterogeneous subset of RNAs that are longer than 200 nucleotides (nt) and transcribed regions without protein-coding potential. Increased advances have shown that many lncRNAs not only are transcriptional “noise,” but also play an important role in numerous biological processes including transcriptional regulation (6, 7), cell cycle and apoptosis (8), and pluripotency and differentiation control (9, 10). Thus, extensive research is required to fully define and integrate lncRNAs into genome biology. Figuring out the role of lncRNAs would better understand the underlying genetic aspects of non-coding RNA on the deposition of blue eggshells.

China has a wide variety of indigenous poultry, with 108 native chicken breeds. The intensive selection for layers producing blue eggshells has been undergoing for a few decades due to demands by consumers. Nevertheless, blue shell eggs show dark blue, light blue, and median color brown-greenish blue, of which brown-greenish shell eggs are especially not found by consumers. Notably, Changshun blue-eggshell chicken is one of the native breeds mainly producing blue eggshell, but a few of them (unselected individuals) also produce brown and pink color eggs. Thus, it is urgent to figure out the underlying genetic mechanism from non-coding RNA and its targeted genes in breeding selection for blue eggshell layers. In the present study, a high-throughput RNA sequencing (RNA-seq) was employed to profile the uterus transcriptome of Changshun blue-eggshell chickens with different eggshell colors (different proportions between protoporphyrin and biliverdin). The aims were to discover and characterize lncRNAs in chicken uterus tissue and identify key genes, lncRNAs, and pathways that are associated with blue eggshell deposition of chickens.

## Materials and Methods

### Animals and Treatments

**Figure 1:**
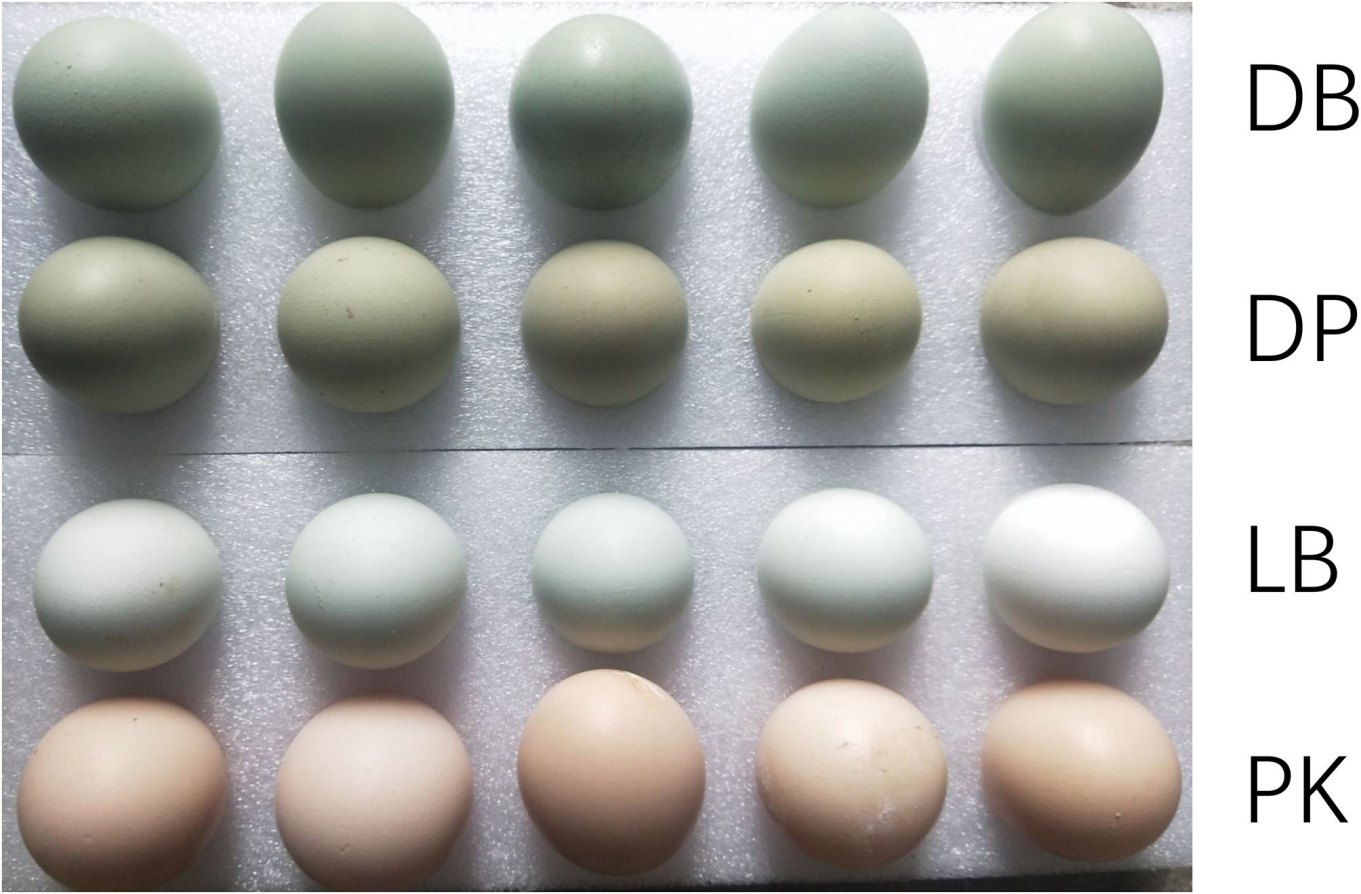
The description of four different eggshell colors of Changshun blue-eggshell chicken. DB, DP, LB, and PK mean chickens producing dark blue, dark brown and greenish, light blue, and pink eggshell eggs, respectively.

The study was approved by the Animal Care Committee of Foshan University (Approval ID: FOSU#080). The experiment was carried out at a breeding farm, Changshun blue eggshell layer, Tiannong Corporation, Guizhou province. A total of 331 layers in a house at 210 days old were observed for 30 days. During these days, three hens stably producing dark blue (DB), four hens producing light blue (LB), and dark brown and greenish (between blue and pink, DP), respectively, were selected for this study (see Figure 1). Besides, four hens producing pink (PK) shell eggs, as a control group, were also involved in the study. Uterine glands from each bird were collected and immediately stored in dry ice and then at −80°C until further processing.

### RNA-seq

Fifteen cDNA libraries were constructed using total uterus RNA. Total RNA was extracted using the RNeasy Mini-Extraction kit according to the manufacturer's instructions (Aidlab, RN2802, China). The 2100 Bioanalyser (Agilent) was used to determine the RNA quality and was further quantified using the ND-2000 (NanoDrop Technologies). Only high-quality RNA sample was used to construct a sequencing library. The relative expression of color deposition-related genes including solute carrier organic anion transporter family member 1C1 (*SLCO1C1*), solute carrier family 16 member 7 (*SLC16A7*), *CD4*, and ST6 N-acetylgalactosaminide alpha-2,6-sialyltransferase 4 (*ST6GALNAC4*) was measured. Primer sequence 5.0 was used to design the primer sequence and synthesized by Shanghai Bioengineering limited company (Supplementary Table 1). The glyceraldehyde-3-phosphate dehydrogenase (*GAPDH*) was selected as a reference gene (11). The purity of RNA was the ratio of OD 260/OD 280 (a measure of protein contamination) in 2.0–2.2, of which 2.2 represented high-quality RNA. The RNA integrity was analyzed by the method of agarose gel electrophoresis, of which the quantity of 28S rRNA was twice than that of 18S rRNA. Then, each qualified RNA sample has reversed the transcript to cDNA using TRUEscript RT MasterMIX in a 20-μl volume containing 1,000 ng RNA and RNase free to 16 μl, 4 μl 5 × TRUE RT MasterMix under the following conditions: 42°C for 10 min (Aidlab, PC5801, China) for RT-qPCR analyses. RT-qPCR was conducted on qTOWER 2.2 touch (Analytik Jena, Germany) in a 20-μl volume containing 10 μl SYBR × Premix Ex Taq (Aidlab, PC3302, China), 0.5 μl of each forward and reverse primer (10 μM), 1 μl of cDNA, and 8 μl ddH_2_O under the following conditions: 95°C for 15 min; 95°C for 10 s, annealing (see Table 1) for 20 s and 72°C for 20 s for 40 cycles. Each amplification was performed for three control replicates and three case replicates. The amplification efficiencies were close to 100%, using the 2^−ΔΔCt^ method for calculating the relative gene expression levels of a sample.

**Table 1:** Differently enriched KEGG pathways with targeted genes of comparisons between chickens with different eggshell colors.

**Comparison**	**Pathways**	***p***	**Targeted genes**
DB vs. DP	Transporters	0.008	*SLC34A1, SLC13A3, SLC35F1, SLC22A6-A, SLC26A1*
	Exosome	0.011	*SLC34A1, GSN, CUBN, DSP, HSPG2, FN1, FLNA*
	Cytoskeleton proteins	0.041	*GSN, DSP, Tpm3, FLNA*
	Cortisol synthesis and secretion	0.028	*Pbx1*
	Apoptosis	0.041	*ITPR2, CASP6*
	Glycerophospholipid metabolism	0.045	*Etnk1*
	Lipid metabolism	0.044	*LPPR4*
DB vs. LB	Transporters	<0.001	*SLC13A3, SLC35F1, SLC25A47, SLC22A6-A, SLC26A1*
	Exosome	0.023	*SLC34A1, CUBN, DSP, ANXA11, COL18A1*
	Cortisol synthesis and secretion	0.025	*Pbx1*
	NOD-like receptor signaling pathway	0.028	*ITPR2, TRAF5*
	Progesterone-mediated oocyte maturation	0.034	*PGR, CPEB2*
	Glycerophospholipid metabolism	0.035	*Etnk1*
DP vs. LB	Inositol phosphate metabolism	0.010	*MTMR1, IPMK*
	Cysteine and methionine metabolism	0.022	*SRM*
	Phosphatidylinositol signaling system	0.024	*MTMR1, IPMK*
	Protein phosphatase and associated proteins	0.031	*MTMR1, IPMK, SSH2, SH3RF1*
PK vs. DB	Transporters	<0.001	*SLCO1C1, SLC16A7, SLC34A1, SLC13A3, SLC25A47, SLC22A4, SLC26A1, OCLN, CNNM2, ABCA4*
	Progesterone-mediated oocyte maturation	0.002	*PGR, BRAF, ANAPC1, CPEB2*
	Ascorbate and aldarate metabolism	0.012	*Ugt1a9*
	Lysosome	0.034	*Cd164*
	General function prediction only	0.039	*ABHD2*
	Glycosyltransferases	0.041	*STT3B, Ganlt16, Ugt1a9, ST8SIA5*
	Butanoate metabolism	0.048	*.-*
PK vs. DP	Transcription factors	0.004	*PGR, FOXP1, Nr6a1, IRF4, Rfx2, Pbx1*
	Transporters	0.006	*SLCO1C1, SLC35F1, SLC4A4, SLC25A47, SLC35F5, SLC22A4*
	Hedgehog signaling pathway	0.007	*GSK3B, PTCH1*
	Other glycan degradation	0.011	*MANBA*
	Lysosome	0.016	*MANBA*
	Nuclear receptors	0.019	*PGR, Nr6a1*
	Cortisol synthesis and secretion	0.027	*Pbx1*
PK vs. LB	Phosphatidylinositol signaling system	0.002	*PI4KA*
	Inositol phosphate metabolism	0.005	*PI4KA*
	Cortisol synthesis and secretion	0.026	*Pbx1*
	Membrane trafficking	0.026	*PI4KA*
	DNA replication proteins	0.039	*FOXP1, Pbx1*

*DB, DP, LB, and PK mean chickens producing dark blue, dark brown and greenish, light blue, and pink eggshell eggs, respectively*.

The total RNA of uterine glands from each was further RNA-seq for mRNA and lncRNA. Strand-specific RNA-seq libraries were generated by TruSeq Stranded Total RNA with Ribo-Zero Gold kit (Illumina, CA, USA) following the manufacturer's recommendations. Sequencing was performed on an Illumina Hiseq 2500 instrument using the TruSeq PE Cluster Kit v3-cBot-HS (Illumina, CA, USA) to generate 150-bp paired-end reads. Quality control and reads statistics were determined by FastQC (0.11.2) (12). Reads containing adapter or poly-N and low-quality reads were discarded, while the remaining clean reads were aligned to the reference chicken genome (Galllus_gallus-5.0) using Hisat (2.0.1) (13). Stringtie (1.2.4) was used to assemble mapped transcripts individually (14), and reference gene annotation was supplied to guide the assembly process. Transcripts from all samples were then merged together with Stringtie merge mode to build a consensus set of transcripts across samples to identify lncRNAs and their nearest-neighbor genes. To reduce the false-positive rates, assembled transcripts were obtained as follows to receive candidate lncRNAs: (1) transcripts with two and above two exons and longer than 200 bp; (2) the reads coverage of transcript more than three was calculated using Stringtie (1.2.4); (3) protein coding potency of transcripts were calculated by three software including coding–non-coding-index (score < 0), coding potential calculator (15) (score < 0), and SwissProt. Transcripts were filtered according to the abovementioned requirements and were considered as candidate lncRNAs and were then blasted to chicken lncRNAs in the ALDB v1.0 database. Reference gene annotation was used to search the nearest-neighboring genes of lncRNAs, and 100 kb was set as the threshold. Differentially expressed lncRNAs were calculated for further prediction, of which those located within the 100-kb distance of the differentially expressed lncRNAs were selected as potential target genes to reduce false positives. The cis role of lncRNAs was on neighboring target genes (16, 17). The quantification of lncRNAs and mRNAs in each sample was calculated by Stringtie. Differentially expressed mRNAs and lncRNAs of uterine glands among different eggshell color chickens were analyzed using the ballgown (2.6.0) R package (18). *p*-value < 0.05 and |fold-change| > 2 were considered as significance threshold. Gene ontology (GO) enrichment and Kyoto Encyclopedia of Genes and Genomes (KEGG) analysis of differentially expressed mRNAs and lncRNAs were carried out by Goatools (https://github.com/tanghaibao/Goatools) and KOBAS (http://kobas.cbi.pku.edu.cn/home.do).

## Results

### Overview of Sequencing and Identification of lncRNA in Chicken Uterus

**Figure 2:**
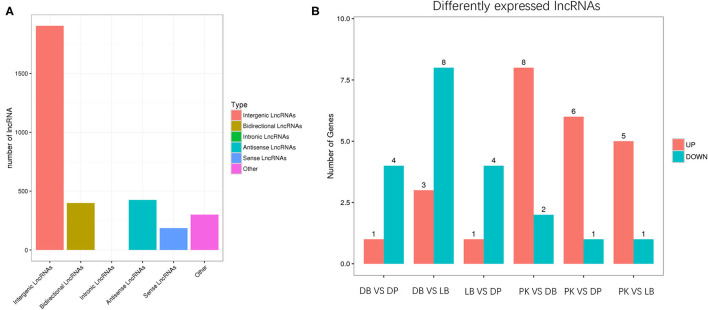
**(A)** represents lncRNAs including intergenic lncRNAs, bidirectional lncRNAs, antisense lncRNAs, sense lncRNAs, and other lncRNAs. **(B)** represents differently expressed lncRNAs between comparisons. DB, DP, LB, and PK mean chickens producing dark blue, dark brown and greenish, light blue, and pink eggshell eggs, respectively.

After quality control, more than 98.2% of the total clean reads with high quality were mapped to Galgal 5.0, and 61,369 assembled transcripts were produced. Detailed information on data quality and mapping statistics is presented in Supplementary Table 2. As a result, 6,275 candidate lncRNAs were captured, with 3,060 known by blasting against the known chicken lncRNAs in ALDB database and 3,215 new lncRNAs. Of the new lncRNAs, there were 1,904 intergenic lncRNAs, 399 bidirectional lncRNAs, 426 antisense lncRNAs, 186 sense lncRNAs, and 300 others (Figure 2).

### Genomic Feature of lncRNAs

The comparison of differently expressed lncRNAs is presented in Supplementary Figure 1B. Between the DB and DP, there were one upregulated and four downregulated genes in the DP group compared to the DB group (*p* < 0.05). Between the DB and LB, there were three upregulated and eight downregulated genes in the LB group compared to the DB group (*p* < 0.05). Between the DP and LB, there were one upregulated and four downregulated genes in the LB group compared to the DP group (*p* < 0.05). We found that as compared to pink-shell eggs, there were eight upregulated and two downregulated genes in dark green-shell eggs, six upregulated and one downregulated gene in green-blue-shell eggs, and five upregulated and one downregulated gene in light green-shell eggs (*p* < 0.05).

There are more known (16,910 on average) and novel mRNAs (14,207 on average) than known (1,430 on average) and novel lncRNAs (2,083 on average) in all the four groups. The heatmaps displayed differentially expressed lncRNAs (Supplementary Figure 1) and mRNAs (Supplementary Figure 2).

### Genomic Feature of mRNAs

**Figure 3:**
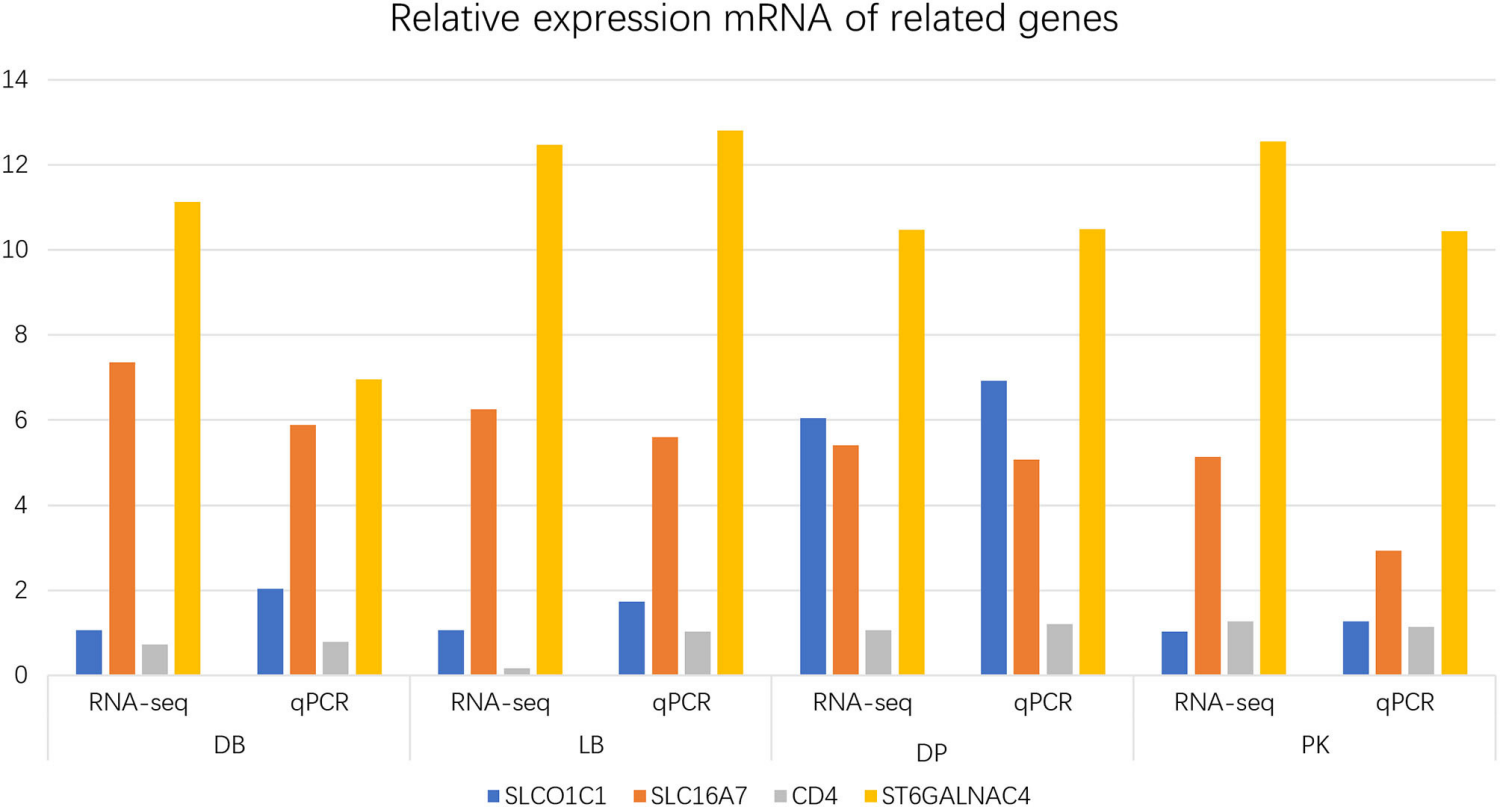
Relative mRNA expression of related genes. DB, DP, LB, and PK mean chickens producing dark blue, dark brown and greenish, light blue, and pink eggshell eggs, respectively.

In relation to the mRNA, 24,102 (79.67%) known transcripts and 199,963 new transcripts were obtained. The differently expressed genes between comparisons are shown in Supplementary Figure 3. Furthermore, the relative foldchange of those selected genes in qPCR was consistent with RNA-seq results, suggesting that the transcript identification and abundance estimation were highly reliable (Figure 3).

There were no significant GO terms between DB and DP. Furthermore, a total of seven significant KEGG pathways including cortisol synthesis and secretion, lipid metabolism, and glycerophospholipid metabolism were obtained (*p* < 0.05, Table 1). SLC family genes were the main targeted genes (*p* < 0.05, Table 1). Besides, gelsolin (*GSN*), cubilin (*CUBN*), desmoplakin (*DSP*), heparan sulfate proteoglycan 2 (*HSPG2*), fibronectin 1 (*FN1*), filamin A (*FLNA*), pre-B-cell leukemia transcription factor 1 (*Pbx1*), inositol 1,4,5-trisphosphate receptor type 2 (*ITPR2*), caspase 6 (*CASP6*), phospholipid phosphatase related 4 (*PLPPR4*), and ethanolamine kinase 1 (*Etnk1*) were also differently enriched between groups, of which the relative expression of *GSN, CUBN, Pbx1*, and *PLPPR4* were upregulated, whereas *DSP, HSPG2, FN1, FLNA, ITPR2, CASP6, Etnk1*, and SLC family genes were downregulated in the former group as compared to the latter group (*p* < 0.01).

Between the comparison of DB vs. LB, the GO term hindbrain tangential cell migration of biological process was significantly enriched, with targeting on gene *PLXNA2* (*p* < 0.05, Supplementary Table 3). Cortisol synthesis and secretion and five other pathways were significantly enriched between the two groups (*p* < 0.05, Table 1). *CUBN, DSP*, Annexin A11 (*ANXA11*), Collagen type XVIII alpha 1 chain (*COL18A1*)*, Pbx1, ITPR2*, TNF receptor associated factor (*TRAF5*), progesterone receptor (*PGR*), cytoplasmic polyadenylation element binding protein 2 (*CPEB2*)*, Etnk1*, and SLC family genes were targeted genes between the two groups, of which the relative expression of SLC family genes, *CUBN, COL18A1, Pbx1, ITPR2, TRAF5, PGR*, and *CPEB2* was upregulated, while that of *DSP, ANXA11*, and *Etnk1* was downregulated in the DB group as compared to the LB group (*p* < 0.05, Table 1).

The GO term of phosphatidylinositol monophosphate phosphatase activity with targeted gene *MTMR1* was found to be enriched between DP and DL (*p* < 0.05, Supplementary Table 3). Inositol phosphate metabolism, cysteine and methionine metabolism, phosphatidylinositol signaling system, and protein phosphatase and associated proteins were identified to be enriched between the two groups (*p* < 0.05, Table 1). Targeted genes myotubularin related protein 1 (*Mtmr1*), inositol polyphosphate multikinase (*IPMK*), spermidine synthase (*SRM*), slingshot protein phosphatase 2 (*SSH2*), and SH3 domain containing ring finger 1 (*SH3RF1*) were identified, and the relative expression of all these genes was upregulated in the DP group compared to the DL group (*p* < 0.05, Table 1).

There were no significant GO terms between the comparison of PK vs. DB. However, seven pathways including transporters, progesterone-mediated oocyte maturation, ascorbate and aldarate metabolism, lysosome, glycosyltransferases, and butanoate metabolism were found to be significantly enriched (*p* < 0.05, Table 1). These pathways were related to targeted genes including SLC family genes, occludin (*Ocln*), adaptor related protein complex 1 gamma 1 subunit (*AP1G1*), ATP binding cassette subfamily A member 4 (*ABCA4*), *PGR*, heat shock protein 90 (*Hsp90*), UDP glycosyltransferase (*UGT*), L-gulono-gamma-lactone oxidase (*GULO*), raf proto-oncogene, serine/threonine kinase (*RAF*), legumain (*LGMN*), anaphase promoting complex subunit 1 (*ANAPC1*), *CPEB, CD164*, abhydrolase domain containing 2 (*ABHD2*), polypeptide N-acetylgalactosaminyltransferase 16 (*Galnt16*), and ST8 alpha-N-acetyl-neuraminide alpha-2,8-sialyltransferase 5 (*ST8SIA5*), of which the relative expression of gene *Ocln, PGR, CPEB, Hsp90, UGT, GULO, Galnt16*, and *ST8SIA5* was upregulated, while *ABCA4, RAF, CD164, LGMN, CPEB*, and SLC family genes were downregulated in the former group as compared to the latter group (*p* < 0.01).

There were eight GO terms and one GO term significantly enriched for cellular components and molecular function between PK and DP (*p* < 0.05, Supplementary Table 2). These GO terms were mainly involved with immune activities including targeted genes ENSGALT00000018840 and TCONS_00059917. Furthermore, transcription factors, transporters, hedgehog signaling pathway, lysosome, nuclear receptors, and cortisol synthesis and secretion were identified to be enriched between the two groups (*p* < 0.05, Table 1). Targeted genes, such as SLC family genes, *PGR*, forkhead box P1 (*FOXP1*), nuclear receptor subfamily 6 group A member 1 (*Nr6a1*), interferon regulatory factor 4 (*IFR4*), *Pbx1*, glycogen synthase kinase 3 alpha (*GSK3B*), patched 1 (*PTCH1*), and mannosidase beta (*MANBA*), were located, of which the relative expression of *Nr6a1, GSK3B, Pbx1*, and *PTCH1* was upregulated, while *PGR, FOXP1, IFR4, MANBA*, and SLC family genes were downregulated in the former group as compared to the latter group (*p* < 0.01).

Between the PK and LB groups, four and three GO terms for molecular function and biological process (*p* < 0.05, Supplementary Table 2) and pathways including the metabolism of protein family and cortisol synthesis and secretion pathways with genes phosphatidylinositol 4-kinase alpha (*PI4KA*), *Pbx1*, and *FOXP1* were significantly enriched (*p* < 0.05, Table 1), of which the relative expression of *Pbx1* was regulated, while *PI4KA* and *FOXP1* were downregulated in the former group as compared to the latter group (*p* < 0.01).

### Interaction Analyses of lncRNAs and mRNA

The RNAplex was used to find the interaction between two long-chain RNA, to predict the complementary binding between antisense/cis lncRNA and mRNA. The program includes the Vienna RNA package and calculates the minimum free energy according to its thermodynamic structure to predict the best base-pairing relationship.

For the comparison of DB vs. DP, there were 153 differentially expressed lncRNAs. ECM–receptor interaction with candidate gene *CD4* binding to lncRNA TCONS_00009117 was identified to be significantly different between groups (*p* < 0.05, Table 2). With respect to cis analysis, 70 targeted lncRNAs were found to be involved in the adjacent protein coding of mRNA. These related mRNAs were significantly enriched on toll-like receptor signaling pathway, retinol metabolism, phagosome, spliceosome, glycosphingolipid biosynthesis-ganglio series, glycerolipid metabolism, glycerophospholipid metabolism, and ECM–receptor interaction different pathways (*p* < 0.05, Table 3). These pathways were mainly enriched on genes toll-like receptor 2 family member A (*TLR2A*), retinol saturas (*RETSAT*), Catenin beta like 1 (*CTNNBL1*), ST6 N-acetylgalactosaminide alpha-2,6-sialyltransferase 4 (*ST6GALNAC4*), lysocardiolipin acyltransferase 1 (*LCLAT1*), and diacylglycerol kinase zeta (*DGKZ*).

**Table 2:** Differently enriched KEGG pathways with targeted genes of lncRNA and mRNA of comparisons between chickens with different eggshell colors.

**Comparison**	**Pathway**	***p***	**lncRNA**	**mRNA**	**Symbol**
DB vs. DP	ECM–receptor interaction	0.040	TCONS_00009117	ENSGALT00000037104	*CD4*
DB vs. LB	Cell adhesion molecules	0.042	TCONS_00009117	ENSGALT00000037104	*CD4*
	RNA transport	0.049	TCONS_00067464	ENSGALT00000008544	*-*
DP vs. LB	Focal adhesion	0.011	TCONS_00002665	ENSGALT00000082154	*VWF*
PK vs. DB	Cytokine–cytokine receptor interaction	0.005	TCONS_00044519	ENSGALT00000000328	*GH*
	Jak–STAT signaling pathway	0.031	TCONS_00044523	ENSGALT00000000328	*GH*
PK vs. DP	Cytokine–cytokine receptor interaction	0.018	TCONS_00034554	ENSGALT00000070014	*CX3CR1*
	SNARE interactions in vesicular transport	0.037	TCONS_00075755	ENSGALT00000082865	*-*
	Neuroactive ligand–receptor interaction	0.038	TCONS_00044519	ENSGALT00000000328	*GH*
	Tryptophan metabolism	0.046	TCONS_00047705	ENSGALT00000016138	*HAAO*
PK vs. LB	ECM–receptor interaction	0.040	TCONS_00000515	ENSGALT00000012832	*LAMB1*
	Spliceosome	0.050	ENSGALT00000029552	ENSGALT00000040616	*ENSGALT00000040616*

*DB, DP, LB, and PK mean chickens producing dark blue, dark brown and greenish, light blue, and pink eggshell eggs, respectively*.

**Table 3:** Differently enriched KEGG pathways with targeted genes of lncRNA of comparisons between chickens with different eggshell colors.

**Comparison**	**Pathway**	***p***	**Targeted genes**	**lncRNA**
DB vs. DP	Toll-like receptor signaling pathway	<0.001	*TLR2A*	TCONS_00055576
	Retinol metabolism	<0.001	*RETSAT*	ENSGALT00000081853
	Phagosome	<0.001	*TLR2A*	TCONS_00055576
	Spliceosome	<0.001	*CTNNBL1*	TCONS_00038047
	Glycosphingolipid biosynthesis-ganglio series	0.002	*ST6GALNAC4*	ENSGALT00000072290
	Glycerophospholipid metabolism	0.045	*LCLAT1, DGKZ*	ENSGALT00000048295
DB vs. LB	TGF-beta signaling pathway	<0.001	*Sp1*	TCONS_00054721
	Glycosphingolipid biosynthesis-ganglio series	<0.001	*ST6GALNAC4*	ENSGALT00000072290
	Pyruvate metabolism	0.002	*ENSGALT00000074835*	ENSGALT00000051137
	Propanoate metabolism	0.003	*ENSGALT00000074835*	ENSGALT00000051137
	Biosynthesis of secondary metabolites	0.016	*LCLAT1*	TCONS_00050772
	Selenocompound metabolism	0.034	*TXNRD1*	TCONS_00008143
	Metabolic pathways	0.039	*ST6GALNAC4, DPM2, LCLAT1*	ENSGALT00000072290
DP vs. LB	Retinol metabolism	<0.001	*RETSAT*	ENSGALT00000081853
	Other types of O-glycan biosynthesis	0.011	*GXYLT1*	ENSGALT00000069802
	Drug metabolism-other enzymes	0.016	*HPRT1, MDM2*	TCONS_00058295
	Cell cycle	0.029	*YWHAE, MDM2*	ENSGALT00000071577
	N-Glycan biosynthesis	0.033	*MAN1C1, ALG1*	TCONS_00040933
	Wnt signaling pathway	0.038	*APC*	TCONS_00040933
	Biosynthesis of secondary metabolites	0.045	*HPRT1, MPI*	ENSGALT00000009843
	p53 signaling pathway	0.046	*MNM2, CASP14*	ENSGALT00000071577
	Neurotrophin signaling pathway	0.046	*-*	ENSGALT00000077105
PK vs. DB	Proteasome	<0.001	*PSMB1/3*	ENSGALT00000088176
	TGF-beta signaling pathway	<0.001	*Sp1*	TCONS_00040436
	Selenocompound metabolism	<0.001	*PAPSS1, TXNRD1*	TCONS_00056173
	Retinol metabolism	<0.001	*TXNRD1, RETSAT*	ENSGALT00000081853
	Sulfur metabolism	<0.001	*RETSAT, TXNRD1*	TCONS_00056173
	Glycosphingolipid biosynthesis-ganglio series	0.003	*RETSAT*	ENSGALT00000072290
	Spliceosome	0.024	*RBMXL1*	ENSGALT00000070508
	Vitamin B6 metabolism	0.025	*PHOSPHO2*	ENSGALT00000085067
	Cell cycle	0.025	*RB1*	ENSGALT00000051325
PK vs. DP	beta-Alanine metabolism	<0.001	*HIBCH*	TCONS_00071353
	p53 signaling pathway	<0.001	*SESN1, CASP14*	ENSGALT00000078895
	Propanoate metabolism	<0.001	*HIBCH*	TCONS_00071353
	RNA degradation	0.001	*XRN1*	ENSGALT00000084047
	Porphyrin and chlorophyll metabolism	0.002	*FECH*	ENSGALT00000087341
	Valine, leucine and isoleucine degradation	0.002	*HIBCH*	TCONS_00071353
	Sulfur metabolism	0.003	*PAPSS1*	TCONS_00056173
PK vs. LB	Retinol metabolism	<0.001	*RETSAT*	ENSGALT00000081853
	N-Glycan biosynthesis	0.003	*MAN1C1, ALG1*	TCONS_00040933
	Cardiac muscle contraction	0.008	*COX6A1*	TCONS_00023198
	Metabolic pathways	0.013	*MAN1C1, ALG1*	TCONS_00040933
	Oxidative phosphorylation	0.014	*COX6A1*	TCONS_00023198
	Protein processing in endoplasmic reticulum	0.032	*MAN1C1, ALG1*	TCONS_00040933
	SNARE interactions in vesicular transport	0.048	*VAMP3*	TCONS_00039175

*DB, DP, LB, and PK mean chickens producing dark blue, dark brown and greenish, light blue, and pink eggshell eggs, respectively*.

Between the comparison of DB vs. LB, 153 differentially expressed lncRNAs were identified, of which 9 lncRNAs were associated with the binding to sense chain of mRNA by antisense analysis. Cell adhesion molecules with targeted gene *CD4* binding to lncRNA TCONS_00009117 and RNA transport pathways were differently identified (*p* < 0.05, Table 2). For cis analysis, 54 targeted lncRNAs were found to be involved in the adjacent protein coding of mRNA. TGF-beta signaling pathway, glycosphingolipid biosynthesis-ganglio series, pyruvate metabolism, propanoate metabolism, biosynthesis of secondary metabolites, selenocompound metabolism, and metabolic pathways were identified to be different between groups (*p* < 0.05, Table 3). These pathways were found to relate with differentially expressed genes including Sp1 transcription factor (*Sp1*)*, ST6GALNAC4, LCLAT1*, and thioredoxin reductase 1 (*TXNRD1*).

Between the comparison of DP vs. LB, there were 224 differentially expressed lncRNAs, of which 18 lncRNAs were associated with the binding to sense chain of mRNA by antisense analysis. Focal adhesion pathway with a candidate gene von Willebrand factor (*VWF*) binding to lncRNA TCONS_00002665 was identified between groups (*p* < 0.05, Table 2). For cis analysis, 115 targeted lncRNAs were found to be involved in the adjacent protein coding of mRNA. These related mRNAs were significantly enriched on 19, 41, and 223 GO terms for cellular components, molecular function, and biological process including pigment metabolic process, respectively (*p* < 0.05). Retinol metabolism, other types of O-glycan biosynthesis, drug metabolism—other enzymes, cell cycle, N-glycan biosynthesis, Wnt signaling pathway, biosynthesis of secondary metabolites, p53 signaling pathway, and neurotrophin signaling pathways were identified (*p* < 0.05, Table 3). *RETSAT*, glucoside xylosyltransferase 1 (*GXYLT1*), hypoxanthine phosphoribosyltransferase 1 (*HPRT1*), MDM2 proto-oncogene (*MDM2*), tyrosine 3-monooxygenase/tryptophan 5-monooxygenase activation protein epsilon (*YWHAE*), mannosidase, alpha, class 1C, member 1 (*MAN1C1*), chitobiosyldiphosphodolichol beta-mannosyltransferase (*ALG1*), *APC*, mannose phosphate isomerase (*MPI*), and caspase 14, apoptosis-related cysteine peptidase (*CASP14)* as candidate genes were identified to be involved with these pathways.

Between the comparison of PK vs. DB, 275 differentially expressed lncRNAs were identified. Furthermore, 23 targeted lncRNAs of antisense with functional annotation information were identified. Environmental information processing of cytokine–cytokine receptor interaction and Jak–STAT signaling pathways with candidate gene *GH* binding to TCONS_00044519 and TCONS_00044523 lncRNAs was identified (*p* < 0.05, Table 2). For cis analysis, 99 targeted genes with functional annotation information were obtained (*p* < 0.05). Besides, proteasome, TGF-beta signaling, vitamin B6 metabolism, selenocompound metabolism, retinol metabolism, sulfur metabolism, glycosphingolipid biosynthesis-ganglio series, spliceosome, and cell cycle pathways with candidate genes including proteasome subunit beta 1/3 (*PSMB1/3*), *Sp1, 3,-*phosphoadenosine 5,-phosphosulfate synthase 1 (*PAPSS1*), *TXNRD1, RETSAT*, RNA binding motif protein, X-linked like 1 (*RBMXL1*), phosphatase, orphan 2 (*PHOSPHO2*), and retinoblastoma 1 (*RB1*) were obtained (*p* < 0.05, Table 3).

Between the comparison of PK vs. DP, there were 368 differentially expressed lncRNAs, of which 34 targeted lncRNAs associated with the binding to sense chain of mRNA by antisense analysis. Cytokine–cytokine receptor interaction, SNARE interactions in vesicular transport, neuroactive ligand–receptor interaction, and tryptophan metabolism pathways with candidate genes C-X3-C motif chemokine receptor 1(*CX3CR1*), *GH*, and 3-hydroxyanthranilate 3,4-dioxygenase (*HAAO*) were significantly identified (*p* < 0.05, Table 2). In relation to cis analysis, 168 targeted lncRNAs were located, having potential roles involving the adjacent protein coding of mRNA. These related mRNAs were significantly enriched on 44, 35, and 57 GO terms for cellular components, molecular function, and biological process, respectively (*p* < 0.05). Beta-alanine metabolism, p53 signaling pathway, propanoate metabolism, RNA degradation, porphyrin and chlorophyll metabolism, valine, leucine, and isoleucine degradation, and sulfur metabolism pathways with 3-hydroxyisobutyryl-CoA hydrolas (*HIBCH*), sestrin 1 (*SESN1*), *CASP14*, 5,-3, exoribonuclease 1 (*XRN1*), ferrochelatase (*FECH*), and *PAPSS1* as candidate genes were significantly identified between groups (*p* < 0.05, Table 3).

Between the comparison of PK vs. LB, 189 differentially expressed lncRNAs were identified, of which 10 lncRNAs were associated with the binding to sense chain of mRNA by antisense analysis. These targeted mRNAs were enriched on 15, 3, and 48 GO for cellular components, molecular function, and biological process, respectively (*p* < 0.05). ECM–receptor interaction and spliceosome different pathways were located (*p* < 0.05, Table 2). For cis analysis, 67 targeted lncRNAs were found to be involved in the adjacent protein coding of mRNA. These related mRNAs were significantly enriched on 44, 35, and 57 GO terms for cellular components, molecular function, and biological process, respectively (*p* < 0.05). Retinol metabolism, N-Glycan biosynthesis, cardiac muscle contraction, oxidative phosphorylation, protein processing in endoplasmic reticulum, fatty acid elongation, biosynthesis of unsaturated fatty acids, and SNARE interactions in vesicular transport were significantly identified between groups (*p* < 0.05, Table 3). Besides, candidate genes *RETSAT*, mannosidase, alpha, class 1C, member 1 (*MAN1C1*), *ALG1*, cytochrome c oxidase subunit 6A1 (*COX6A1*), and vesicle-associated membrane protein 3 (*VAMP3*) were identified between groups.

## Discussion

### Profiling of the mRNA Sequencing

With respect to the coding proteins, we found that different GO terms between pink-eggshell and blue-eggshell chickens were mainly targeting immune- and transporter-related terms with SLC family, *IgJ*, CD family, topoisomerase (DNA) III beta (*Top3b*), and *MTMR* genes. Our results again provide evidence of the candidate gene *SLCO1B3* for a causative mutation on pigment deposition of the blue eggshell phenotype (5) and also an immune relative implication. Within those blue-eggshell chickens, the GO terms hindbrain tangential cell migration and phosphatidylinositol monophosphate phosphatase activity with targeted gene *Plxna2* and *Mtmr1* were specifically identified in dark blue-eggshell chickens. Both the *Plxna2* and *Mtmr1* are known to refer to nerve systems (19) (Pasterkamp), which indicates divergence of nerve function among different degrees of blue-eggshell chickens.

In relation to the KEGG pathway, the lysosome pathway targeted with immune-related genes *CD164* and *IRF4* was significantly enriched in the DB as compared to the PK group. Protoporphyrin IX, as a precursor of heme, is ubiquitously present in all living cells in small amounts, which is involved with inflammation (20). The zinc protoporphyrin disodium (Zn-PP-2Na) is demonstrated to have anti-inflammatory properties by inhibiting type II collagen-induced arthritis in mice (21). In an overview of the differently enriched pathways, we found that several pathways of the comparison of PK vs. DP coincided with both the comparison of PK vs. DB and PK vs. LB. Besides, each comparison has its pathways varied among other comparisons. These indicate that different genetic mechanisms of chickens with different eggshell colors and the median color between pink and blue did, to some extent, overlap both the two biological functions of pink- and dark blue-eggshell chickens. This finding is also evidenced by the current results that showed that the relative expression of the SLC family is higher in the DB and the DP groups than in the PK group, and in the DB group than in the DP and the LB groups. Notably, targeted SLC family-related genes were not different between the comparison of chickens producing light blue eggshell eggs and pink shell eggs, as well as chickens with light blue shell and brown-blue shell eggs. These imply that the mechanism of deposition of blue pigment is different between the light and dark blue eggshell, of which the deposition of dark and brown-greenish blue is involved with the SLC family gene, but not the light blue pigment.

Clearly, the transporter pathway is mainly targeting SLC family genes and is involved with chickens producing pink and light/brown eggs. The progesterone-mediated oocyte maturation pathway with targeted gene *PGR* was significantly enriched in dark eggshell chickens as compared to light and pink-eggshell chickens. This implies that the progesterone may be involved with the pigment deposition when oviposition occurs. The cuticle is related to the deposition of, or contains to some extent, the pigment deposition of the brown eggshell pigment (22). Indeed, the progesterone, as a factor controlling cuticle deposition, is also related to pigment deposition (23). This leads to an explanation that shell strength associated with cuticle deposition increased as the darkness of the shell increased (24). Besides, the cortisol synthesis and secretion pathway and the relative expression of targeted gene *Pbx1* upregulated with the darkness of the shell decreased. It is generally known that the cuticle and pigment deposition is affected by a mild environmental stressor that further causes temporary inhibition of the reproductive axis and an increase in circulating corticosteroids (25). A previous study demonstrated that environmental contamination (one of the environmental stressors) negatively correlated with the blue-green chroma (26). These indicate that the increased stress may be associated with the deceased coloration of eggshell, but more work is needed to investigate what induces its release.

### Profiling of the lncRNA Sequencing

LncRNAs in the chicken genome exhibit similar features to those reported in other species, for instance, a significant expression correlation with adjacent protein-coding genes and a high level of tissue specificity. Enrichment analyses of lncRNA-adjacent protein-coding genes also show that chicken lncRNAs likely regulate transcription, cell proliferation, apoptosis, and development (27). In this study, we consider that the potential of lncRNAs would advance our understanding of the genetic mechanisms underlying the trait of pigment deposition of layers. Our lncRNA results showed low expression, shorter transcript length, and fewer exons among species as compared to mRNA, which agree with the results of previous studies and indicate that the lncRNAs identified here were reliable (28, 29). In an overview, we found some enriched pathways such as cell cycle related to lncRNAs.

Significantly different lncRNAs were associated with the binding to sense chain of mRNA by antisense analysis, of which those mRNAs were further used for GO term and KEGG analysis. As compared to the profiling of mRNA sequencing, there are relatively fewer GO terms and pathways relating to targeted lncRNAs associating with mRNAs. It is likely that the significant pathways between comparisons of PK and different blue shell chickens were mainly on immune- and nerve-related pathways, which is consistent with the above mRNA profiling. The pathway cytokine–cytokine receptor interaction is known to have effects on the activation of interleukin-3 (30), while the Jak–STAT signaling pathway is involved with innate immune response in invertebrates (31) and regulates the gene expression of IL-6 and IL-10 (32). Besides, SNARE proteins are known to be linked to exocytosis of insulin granules in β cells (33). The tryptophan metabolism is associated with the body discoloration of crustacea (34) and the regulation of pigment synthesis in Malassezia furfur (35). Notably, the dysfunction of tryptophan metabolism is relative to neurodegenerative diseases, such as tuberculosis (36) and schizophrenia (37). This agrees with the role of the transcript profiling of coding protein on nerve-related function between chickens producing pink and blue shell eggs. These pathways were targeted on *GH, CX3CR1, HAAO, LAMB1*, and ENSGALT00000040616 binding to several lncRNAs including ENSGALT00000000328, ENSGALT00000070014, ENSGALT00000082865, ENSGALT00000016138, ENSGALT00000012832, and ENSGALT00000040616 by antisense analysis, which indicates that these lncRNAs may be involved with the base pairing, gene silencing, and transcription and stability of those binding mRNA. Interestingly, except for some pathways relative to lipids and amino acid metabolisms, porphyrin and chlorophyll metabolism with targeted gene *FECH* and oxidative phosphorylation and cardiac muscle contraction pathways with targeted gene *COX6A1* were found between pink-eggshell and blue-eggshell chickens. These pathways and genes are known to be linked to the color pigments, which implies that lncRNAs also play an important role in affecting adjacent protein coding of mRNAs.

LncRNAs including TCONS_00009117, TCONS_00067464, and TCONS_00002665 were identified to be involved with the eggshell color deposition. These genes and lncRNAs are relative to ECM–receptor interaction, cell adhesion molecules, RNA transport, and focal adhesion pathways. Among chickens producing different blue color eggs, targeted genes *CD4* and *VWF* are located. The immune-related gene found between dark blue-eggshell chickens and brown greenish eggshell chickens are also supported by the cis analysis that shows that the toll-like receptor signaling pathway with the TLR family gene that serves as an immune-related indicator (38) was enriched between the two groups. Among the comparisons of the three blue shell chickens, most pathways were mainly enriched on lipid-related metabolisms, such as retinol metabolism, pyruvate metabolism, propanoate metabolism, O-glycan biosynthesis, N-Glycan biosynthesis, glycerophospholipid metabolism, and glycosphingolipid biosynthesis-ganglio series pathways. The glycerophospholipid metabolism in both the protein-coding and non-coding genes related to pathways was found between chickens producing different degrees of blue eggshell color. The glycerophospholipid metabolism has predictive value on acute graft vs. host disease (1). The disturbance of hippocampal glycerophospholipid metabolism may result in an imbalance of hippocampal sphingolipid and glycerophospholipid metabolism (39). These literatures somehow indicate that the dark blue color may play a protective role in bacterial infection to animals, but further investigation is needed to explain the interaction between lipid-related metabolisms and blue pigment deposition. The glycerophospholipid-related pathways are also found in mRNA sequencing, suggesting that the lncRNAs (ENSGALT00000072290) exerted similar functions in color formation by modulating the adjacent proteins. Accordingly, *Plxna2, Mtmr1,Pbx1, PGR, FECH, COX6A1, CD4*, and *VWF* may be key candidate genes, and ENSGALT00000000328, ENSGALT00000070014, ENSGALT00000082865, ENSGALT00000016138, ENSGALT00000012832, ENSGALT00000040616, TCONS_00009117, TCONS_00067464, and TCONS_00002665 are key lncRNAs related to the blue color deposition.

## Conclusion

In conclusion, transcriptome sequencing was first used in this study to generate the expression profile of lncRNAs and mRNAs in the chicken uterus. Integrating analysis of lncRNA and mRNA profiles of most pathways was mainly enriched on lipid-related metabolisms as found in mRNA sequencing. The lncRNAs exerted similar functions in color formation by modulating, including pigment disposition and immune- and lipid-related metabolisms. Our results provide a catalog of chicken uterine lncRNAs and genes worthy of further studies to understand their roles in selection for blue eggshell color layers.

## Data Availability Statement

The datasets presented in this study can be found in online repositories. The names of the repository/repositories and accession number(s) can be found at: NCBI [accession: PRJNA746050].

## Ethics Statement

The animal study was reviewed and approved by the Animal Care Committee of Foshan University (Approval ID: FOSU#080).

## Author Contributions

HL obtained the funding and designed this project. SC, KC, FL, JD, ZM, JX, GL, and HL performed the experiment. SC, KC, and ZM analyzed and interpreted the data. SC, KC, and HL drafted and revised the manuscript. All authors came to an agreement for publication.

## Funding

This work was supported by the Guangdong Provincial Key Laboratory of Animal Molecular Design and Precise Breeding (2019B030301010), the Key Laboratory of Animal Molecular Design and Precise Breeding of Guangdong Higher Education Institutes (2019KSYS011), and the Germplasm Improvement Talent Base in Guizhou Changshun Tiannong Green Shell Laying Hen Industrial Co. Ltd. The funder was not involved in the study design, collection, analysis, interpretation of data, the writing of this article, or the decision to submit it for publication.

## Conflict of Interest

FL and JD are employed by Guizhou Changshun Tiannong Green Shell Laying Hen Industrial Co. Ltd. The remaining authors declare that the research was conducted in the absence of any commercial or financial relationships that could be construed as a potential conflict of interest.

## Publisher's Note

All claims expressed in this article are solely those of the authors and do not necessarily represent those of their affiliated organizations, or those of the publisher, the editors and the reviewers. Any product that may be evaluated in this article, or claim that may be made by its manufacturer, is not guaranteed or endorsed by the publisher.
