# Supplementary material for: Insights Into mRNA and Long Non-coding RNA Profiling RNA Sequencing in Uterus of Chickens With Pink and Blue Eggshell Colors
Source: Front Vet Sci. 2021 Oct 7;8:736387. doi: 10.3389/fvets.2021.736387 (PMC8529033; doi:10.3389/fvets.2021.736387)
Supplement: Supplementary file 1 [file Data_Sheet_1.pdf]

Table S1. Primer sequences for the target mRNAs used for PCR amplification

Genes	Sequence	Product (bp)
SLCO1C1	F: GTGAGAACTCGGATGTAGC	19
	R: ACGCAGAAATGTAGGTGAT	19
SLC16A7	F: GTTTCCCAAAGCCATCACG	19
	R: CCCAGCCACACAGCAGTTC	19
CD4	F:GACAGAAACACAGAGGGAGGAA	22
	R:CCAAGATGACCGCAAACACA	20
ST6GALNAC4	F:GGGATAAAGATGCTGGTC	18
	R:GAAGAAGTAGGGCTGGTT	18

Table S2. The detailed information of data quality and mapping statistics

Sample	Total Reads	Unmapped Reads	Unique Mapped Reads	Multiple Mapped reads	Mapping Ratio
DB1	66933846	9186723 (13.73%)	56878313 (84.98%)	868810 (1.30%)	86.27%
DB2	71235998	9324017 (13.09%)	61187943 (85.89%)	724038 (1.02%)	86.91%
DB3	58969582	8496603 (14.41%)	49768877 (84.40%)	704102 (1.19%)	85.59%
LB1	65521016	9011746 (13.75%)	55668002 (84.96%)	841268 (1.28%)	86.25%
LB2	60752168	8359787 (13.76%)	51743367 (85.17%)	649014 (1.07%)	86.24%
LB3	63518722	8335613 (13.12%)	54435275 (85.70%)	747834 (1.18%)	86.88%
LB4	61232432	9180970 (14.99%)	51312050 (83.80%)	739412 (1.21%)	85.01%
DP1	69784876	9774419 (14.01%)	59122971 (84.72%)	887486 (1.27%)	85.99%
DP2	75253212	21866255 (29.06%)	52719845 (70.06%)	667112 (0.89%)	70.94%
DP3	65389756	12174866 (18.62%)	52378812 (80.10%)	836078 (1.28%)	81.38%
DP4	58000214	11372594 (19.61%)	45896172 (79.13%)	731448 (1.26%)	80.39%
PK1	61140464	10326076 (16.89%)	50098114 (81.94%)	716274 (1.17%)	83.11%
PK2	65872970	8866775 (13.46%)	55864711 (84.81%)	1141484 (1.73%)	86.54%
PK3	72096974	9926466 (13.77%)	61101692 (84.75%)	1068816 (1.48%)	86.23%
PK4	65492686	8981299 (13.71%)	55563203 (84.84%)	948184 (1.45%)	86.29%

DB, DP, LB and PK mean chickens producing dark blue, dark brown and greenish, light blue, and pink eggshell eggs, respectively.

Table S3 Gene ontology with targeted genes of comparisons between chickens producing eggshell of different shell colors

Comparisons	Gene ontology	P	Genes
DB vs DP	None		
DB vs LB	P-Hindbrain tangential cell migration	<0.001	*TCONS_00044210; TCONS_00044211*
DBG vs LB	F-Phosphatidylinositol monophosphate phosphatase activity	<0.001	*TCONS_00058846; TCONS_00058849*
PK vs DB	None		
PK vs DP	C-Immunoglobulin complex, circulating	<0.001	*ENSGALT00000018840; TCONS_00059917*
	IgA/M immunoglobulin complex/circulating	<0.001	*ENSGALT00000018840; TCONS_00059917*
	C-Polymeric IgA immunoglobulin complex	<0.001	*ENSGALT00000018840; TCONS_00059917*
	C-Dimeric IgA immunoglobulin complex	<0.001	*ENSGALT00000018840; TCONS_00059917*
	C-Immunoglobulin complex	<0.001	*ENSGALT00000018840; TCONS_00059917*
	F-Immunoglobulin binding	<0.001	*ENSGALT00000018840; TCONS_00059917*
PK vs LB	F-Tricarboxylic acid transmembrane transporter activity	<0.001	*ENSGALT00000009642; TCONS_00028361*
	F-C4-dicarboxylate transmembrane transporter activity	<0.001	*ENSGALT00000009642; TCONS_00028361*
	F-DNA topoisomerase activity	<0.001	*TCONS_00022567; TCONS_00022569*
	F-Phosphatidylinositol monophosphate phosphatase activity	0.001	*TCONS_00058847; TCONS_00058849*
	P-Succinate transport	<0.001	
	P-Tricarboxylic acid transport	<0.001	
	P-Rrespiratory chain complex III assembly	<0.001	

DB, DP, LB and PK mean chickens producing dark blue, dark brown and greenish, light blue, and pink eggshell eggs, respectively. C, F, and P represent Cellular Component, Molecular Function, and Biological Process, respectively.

Figure S1

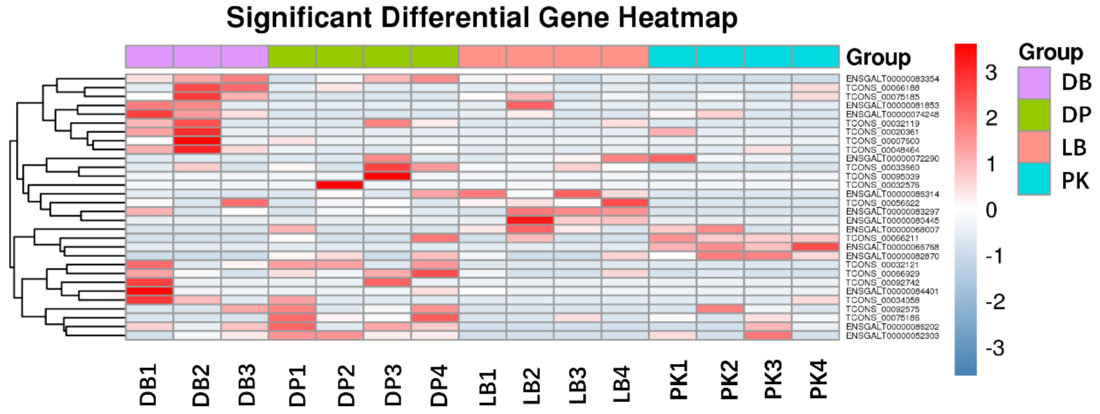

Figure S2: Heatmaps of differentially expressed lncRNA. DB, DP, LB and PK mean chickens producing dark blue, dark brown and greenish, light blue, and pink eggshell eggs, respectively.

Figure S2

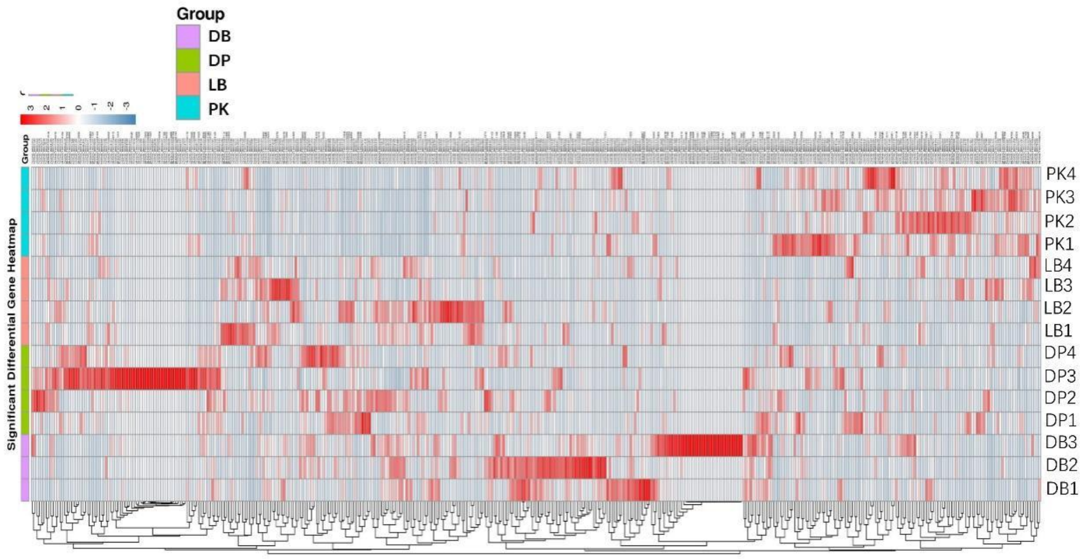

Figure S3: Heatmaps of differentially expressed mRNA. DB, DP, LB and PK mean chickens producing dark blue, dark brown and greenish, light blue, and pink eggshell eggs, respectively.

Figure S3

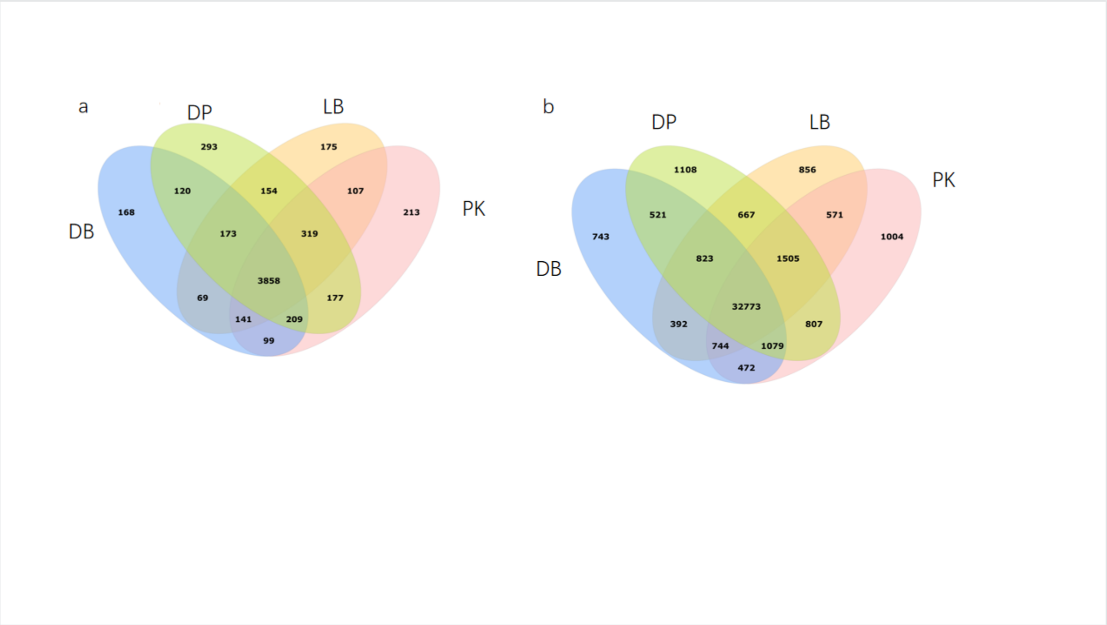

Figure S3: a represents lncRNAs and mRNA of Venn. DB, DP, LB and PK mean chickens producing dark blue, dark brown and greenish, light blue, and pink eggshell eggs, respectively.

Figure S4

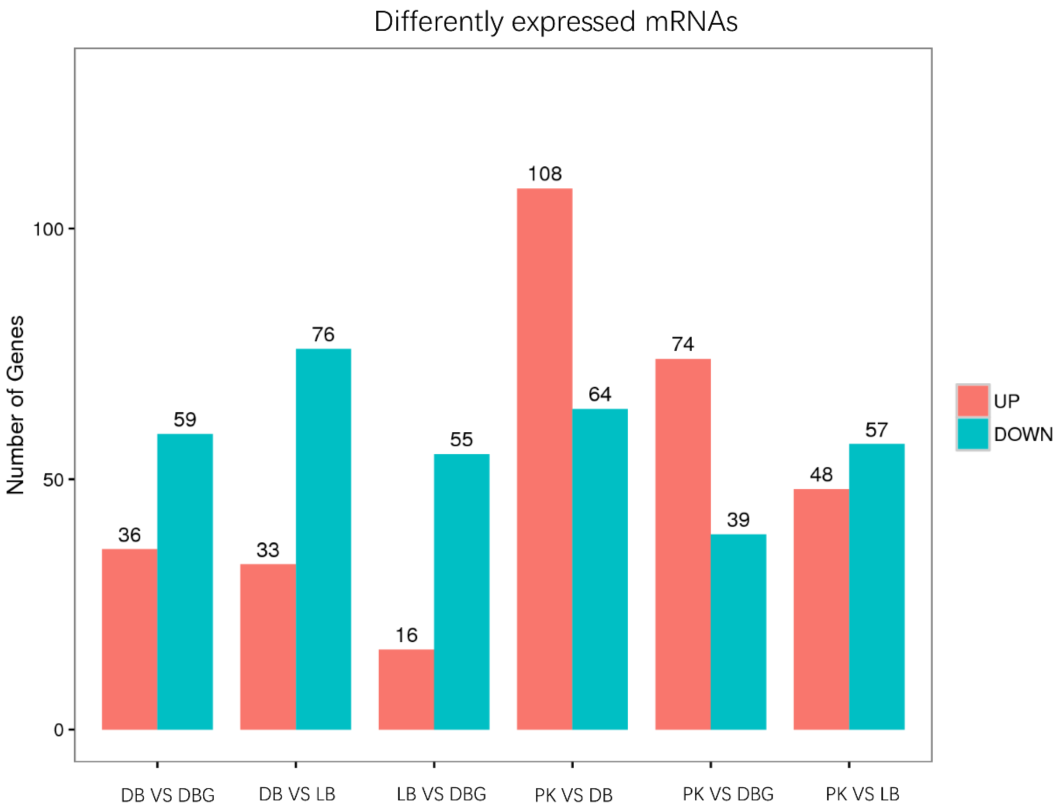

Figure S4: Differently expressed genes between comparisons. DB, DP, LB and PK mean chickens producing dark blue, dark brown and greenish, light blue, and pink eggshell eggs, respectively.
